# Orthotic Insoles Improve Gait Symmetry and Reduce Immediate Pain in Subjects With Mild Leg Length Discrepancy

**DOI:** 10.3389/fspor.2020.579152

**Published:** 2020-12-16

**Authors:** Charlotte Menez, Maxime L'Hermette, Jeremy Coquart

**Affiliations:** ^1^Normandie Univ, UNIROUEN, CETAPS, Rouen, France; ^2^Orthodynamica Center, Mathilde Hospital 2, Rouen, France

**Keywords:** leg length inequality, gait analysis, foot orthoses, musculo skeletal diseases, podiatry, walking

## Abstract

**Background:** Mild leg length discrepancy can lead to musculoskeletal disorders; however, the magnitude starting from which leg length discrepancy alters the biomechanics of gait or benefits from treatment interventions is not clear.

**Research question:** The aim of the current study was to examine the immediate effects of orthotic insoles on gait symmetry and pain on mild leg length discrepancy according to two groups of the leg length discrepancy (i.e., LLD ≤ 1 cm vs. LLD > 1 cm).

**Methods:** Forty-six adults with mild leg length discrepancy were retrospectively included and classified into two groups (G_LLD≤1cm_ or G_LLD>1cm_). All subjects underwent routine 3D gait analysis with and without orthotic insoles. The symmetry index was calculated to assess changes in gait symmetry between the right and left limbs. Pain was rated without (in standing) and with the orthotic insoles (after 30 min of use) on a visual analog scale.

**Results:** There was a significant improvement in the symmetry index of the pelvis in the frontal plane (*p* = 0.001) and the ankle in the sagittal plane (*p* = 0.010) in the stance with the orthotic insoles independent from the group. Pain reduced significantly with the orthotic insoles independently from the group (*p* < 0.001).

**Significance:** Orthotic insoles significantly improved gait symmetry in the pelvis in the frontal plane and the ankle in the sagittal plane, as well as pain in all subjects (both LLD ≤ 1 cm and LLD > 1 cm) suggesting that it may be appropriate to treat even mild leg length discrepancy.

## Introduction

Leg length discrepancy (LLD) can be either caused by anatomical deformities originating from true differences in the bony structures of the lower limb, or it may be functional, resulting from abnormal lower limb movements (Khamis and Carmeli, 2018). The diagnosis (Brady et al., 2003), classification (Gurney, 2002), and treatment (Campbell et al., 2018) of LLD remain controversial among both researchers and clinicians. Some authors classify discrepancies ≤2.0 cm as mild (Moseley, 1996), while others consider discrepancies of up to 3.0 cm as mild (Reid and Smith, 1984; McCaw and Bates, 1991; Gurney, 2002; Brady et al., 2003; Campbell et al., 2018). These classifications are intended to guide practitioners in the treatment of LLD, but there is much disagreement in the literature as to the magnitude from which LLD requires treatment. It has been suggested that orthotic insoles (OIs), shoe lifts, or other clinical interventions to equalize leg length should be considered for LLD ≥ 1.0 cm (White et al., 2004), or even between 0.5 and 1.0 cm (Khamis and Carmeli, 2018). However, other authors are more conservative, suggesting that below 2 cm, no treatment is required (Moseley, 1996).

The lack of consensus regarding the need to treat mild LLD stems from the fact that there is no real agreement as to the biomechanical effects of a mild LLD on lower limb and spinal joints during walking (Friberg, 1982; Kaufman et al., 1996; Goel et al., 1997; Resende et al., 2016; Khamis and Carmeli, 2018). Many studies (Friberg, 1984; Walsh et al., 2000; Seeley et al., 2010; Murray and Azari, 2015; Resende et al., 2016; Tallroth et al., 2017) have reported that even mild LLD can cause lower limb biomechanical disorders. For example, one study (Walsh et al., 2000) found that compensatory strategies and asymmetrical gait occurred from 1.0 cm of LLD induced by foot lifts (from 1 to 5 cm high). Similar results were reported in an earlier study (Kaufman et al., 1996) in which the authors also hypothesized that individuals with even mild LLD use compensatory functional mechanisms to attenuate the effect of the LLD, presumably to minimize displacement of the center of body mass. However, the effect of mild LLD on gait has not been unequivocally demonstrated (Resende et al., 2016; Khamis and Carmeli, 2018).

Mild LLD, including LLD ≤ 1 cm, has been associated with an increased risk of knee osteoarthritis (Harvey, 2010) and scoliosis (Specht and De, 1991), both of which are frequently associated with low back pain (Defrin et al., 2005). Mild LLD is therefore frequently treated with the aim of preventing the development of such secondary pathologies. OIs are the most frequently used treatment (Kendall et al., 2014) likely because they are noninvasive, inexpensive, and readily available (Defrin et al., 2005). Despite the widespread use of OI, their impact on gait kinematics (Bandy and Sinning, 1986; Goel et al., 1997; Bangerter et al., 2019) and pain (Defrin et al., 2005; Golightly et al., 2007) has been little studied in subjects with mild anatomical LLD. Recently, Menez et al. (2020) evaluated the effect of OI on gait kinematics and low back pain in subjects with mild LLD. They found that changes in gait symmetry varied and was specific across individuals; however, low back pain decreased in all subjects after the use of OI. However, mild LLD is commonly not treated in patients with low back pain (Junk et al., 1992; Mannello, 1992; Defrin et al., 2005). Moreover, mild LLD is frequently found in the adult population (Junk et al., 1992; Mannello, 1992), and the correction of LLD ≤ 1 cm remains insufficiently incorporated into the treatment of low back pain (Junk et al., 1992; Mannello, 1992; Defrin et al., 2005), with many clinicians continuing to overlook the potential impact of mild LLD (Defrin et al., 2005). There is disagreement about the correct treatment and the magnitude of LLD (Gurney, 2002). Indeed, for White et al. (2004), OIs to equalize leg length should be considered in subjects with LLD ≥ 1 cm, whereas Khamis and Carmeli (2018) go further, suggesting that even mild LLD between 0.5 and 1 cm should be treated. This recent position of Khamis and Carmeli (2018) is in contradiction with other previous studies suggesting that mild LLD is naturally compensated and should therefore be neglected without any treatment being considered. Apart from the definite interest on pain, the evidence still appears to be limited in terms of kinematics. Therefore, we have searched for additional information to support the interest or not to treat real LLD ≤ 1.0 cm.

Studies are therefore needed to clearly identify the magnitude of LLD from which OI improves gait kinematics and/or pain.

It seems that LLD causes asymmetry in the locomotion of the lower limbs, leading to pain, with a disruption of normal biomechanical function. The functional alterations increase biomechanical disorders, asymmetrical gait, low back pain, and/or other pain, and may even promote the development of associated pathologies such as osteoarthritis of the hip or knee. OI is a treatment often used in podiatry to try to reduce biomechanical asymmetries and pain. We hypothesize that OI can reduce the asymmetries and associated pain in subjects with mild and very mild LLD during walking.

The primary aim of this study was therefore to evaluate the immediate effects of OI on gait symmetry and pain according to the degree of mild LLD (i.e., LLD ≤ 1 cm vs. LLD > 1 cm <3 cm). The secondary aim was to analyze the specific effects of OI on lower limb joint kinematics.

## Materials and Methods

### Subjects

This 18-month, retrospective study, included data from consecutive patients with mild LLD followed with a prescription for OI from their General Practitioner. The study was written according to the STrengthening the Reporting of OBservational studies in Epidemiology (STROBE) statement (Von Elm et al., 2014). Data from all patients meeting the following criteria were analyzed retrospectively. Only adults (aged between 18 and 70 years) were included. None previously had correction of their LLD. The diagnosis of mild LLD (≤3.0 cm) was confirmed by a chiropodist using an accepted clinical procedure (Khamis and Carmeli, 2017). The cutoff of 3.0 cm was selected according to Campbell et al. (2018). Subjects were excluded if they were obese (body mass index ≥ 30 ^*^kg m^−2^) or if they had a history of surgery, lower limb injury, or neuromuscular or vascular pathology in the last 6 months (information routinely collected during the clinical examination). Subjects were classified into one of two groups, according to the magnitude of the LLD: G_LLD ≤ 1cm_ and G_LLD>1cm_ in line with the studies of Seeley et al. (2010) and Defrin et al. (2005).

All the subjects included had undergone routine care, including biomechanical gait analysis with and without the OI as is the usual procedure in our center.

All procedures performed in this study were in accordance with the ethical standards of the institutional and national research committee and with the 1964 Helsinki declaration and its later amendments, and written informed consent was obtained from each subject (Ethical committee number: IRB00012476-2020-15-07-61).

### Procedure

The chiropodist performed a clinical examination that included measurement of the LLD, rating of pain, and collection of sociodemographical and anthropometrical data (i.e., sex, age, body mass, height, and body mass index).

LLD was measured using a direct clinical method (mean of two measurements of the distance between the anterior superior iliac spine and the medial malleolus, while lying in a supine position, using a tape measure). This direct method has already been shown to be valid and reliable in comparison with computed tomography scan (Jamaluddin et al., 2011; Neelly et al., 2013). The mean of three measures was used (Beattie et al., 1990). For this study, the intra-tester reproducibility for the measurement with the tape measure was good, with an ICC of 0.809.

Subjects were referred by a physician for causes of acute muscular affection in low back or lower limb. Even if in this study all causes of pain were retained, we were only interested in one pain per subject with LLD (the most painful condition). The origin of the main cause of pain was investigated, and its intensity was assessed using a visual analog scale. Subjects were asked to stand for 5 min (Golightly et al., 2007) and then to rate their immediate pain on a visual analog scale graded from 0 (no pain) to 10 (maximal pain), as proposed by Hayashi et al. (2015). The location of pain was noted for each subject (low back, hip, knee, or ankle). The average of all these pains was calculated without and with the OI.

**Figure 1:**
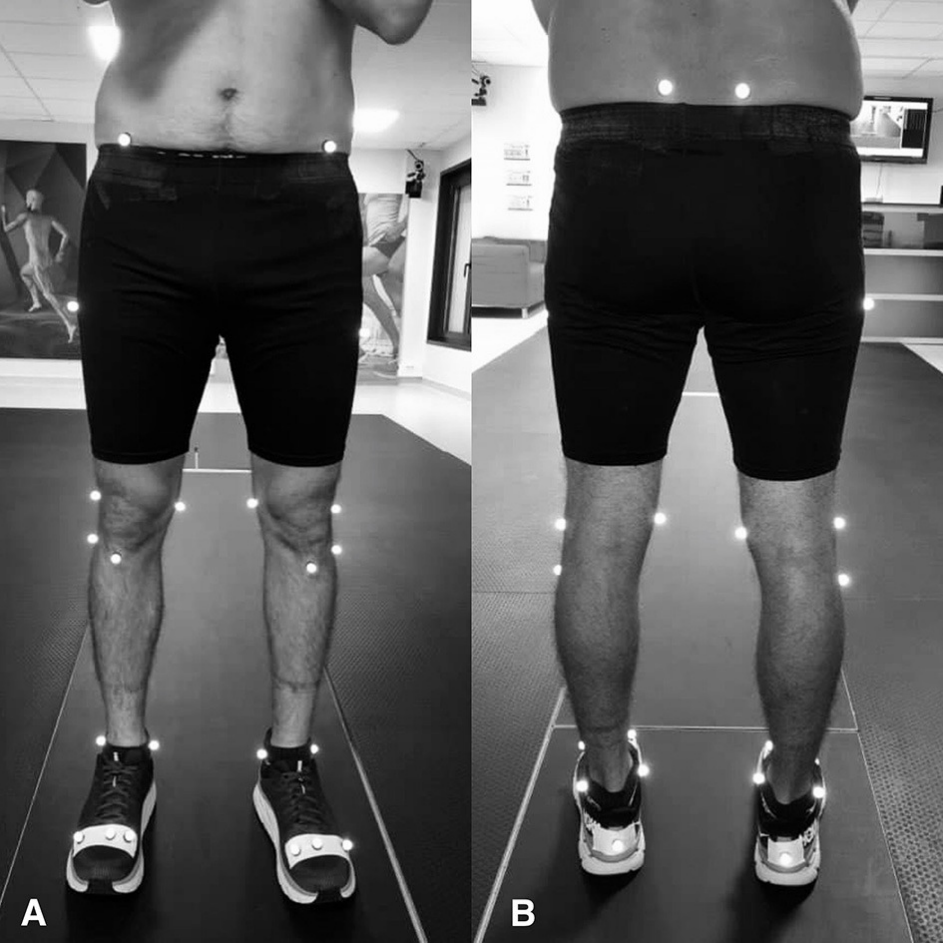
Istituti Ortopedici Rizzoli (IOR) lower body marker set—anterior **(A)** and posterior **(B)** views (Leardini et al., 2007).

Gait analysis was then performed using a Qualisys pro-reflex motion analysis system (Qualisys AB®, Göteborg, Sweden) with 10 infrared video cameras at a sampling frequency of 120 Hz. Twenty reflective markers were fixed to the anatomical landmarks: the two most anterior and the two most posterior margins of the iliac spines, the most lateral prominence of the greater trochanter and of the lateral femoral epicondyle, the proximal tip of the head of the fibula, the most anterior border of the tibial tuberosity, the lateral prominence of the lateral malleolus, the Achilles tendon insertion on the calcaneous, and the dorsal margins of the 1st and 5th metatarsal heads, in accordance with Leardini et al. (2007) (Figure 1). The same investigator positioned the markers on all subjects. A static calibration was carried out before the gait trials in order to generate a neutrally aligned reference “*IOR lower body*” model with respect to the coordinate system of each segment. Subjects wore their usual shoes without OI, which was necessary because the aim was to put them in a walking condition to which they are accustomed. After 10 min of familiarization with the environment by walking around the room, the subjects were instructed to walk at a self-selected speed along the 15 m walkway. Four trials were recorded, and gait cycles performed in the center 10 m of the walkway were used in the analysis. Each trial consisted of five gait cycles making a total of 20 gait cycles for each subject.

**Figure 2:**
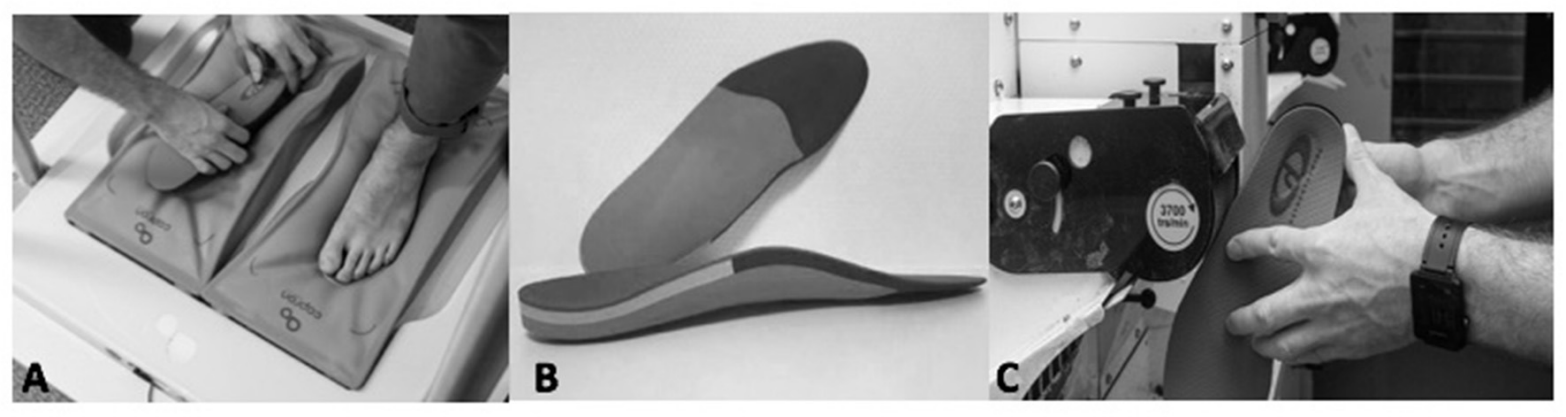
Thermoforming the orthotic insoles **(A)**. Orthotic insoles in ethinyl vinyl acetate, resin, and polyethylene **(B)**. Further shaping the orthotic insoles **(C)**.

The chiropodist made the OI using a thermoforming process: the OIs were first warmed before being molded using a pillow mold to obtain the foot imprint, as recently described by Menez et al. (2020). The materials used in the OIs were ethinyl vinyl acetate, resin, and polyethylene. Once the OIs were molded, they were further shaped to effectively counteract the effects of the LLD and rebalance the kinematics of walking. The whole procedure took 30 min. They were made according to the therapeutic needs of the subjects, with a heel lift incorporated into the OI of the short leg. The heel lifts were partially corrective of the LLD, to 50%, and were shaped from the calcaneus to the Chopart joint (Figure 2). This corrective strategy is used empirically by the pedicurist-chiropodist for all subjects with LLD. At the end of the process, the pedicurist-chiropodist checked the impact of the OI by examining the iliac crest position in the frontal plane while the subject was standing.

The subjects then wore the OIs for 30 min during which time they walked within the center (familiarization phase). After this time, the kinematic analysis and pain rating were repeated with the OI.

The 3D displacement of the markers was processed, and kinematic variables were calculated using Visual3D software (C-Motion®, Germantown, United States) with inverse kinematics approach. The anatomical reference frames for each segment were defined according to Cappozzo et al. (1995), consistent with the international recommendations (Wu and Cavanagh, 1995; Wu et al., 2002). Standard coordinate systems (Grood and Suntay, 1983) were attributed to each joint. Joint angles were defined by rotations occurring about the three joint coordinate axes. For the hip and knee joints, flexion/extension was defined as the relative rotation about a fixed medio-lateral axis (Z), internal/external rotation as the relative rotation about a fixed vertical axis (Y), and abduction/adduction about a “floating” anterior–posterior axis (X). For the ankle joint, these three rotations were defined, respectively, as dorsiflexion/plantarflexion, inversion/eversion, and abduction/adduction. Pelvic tilt (anterior–posterior), rotation, and obliquity (lateral tilt) were calculated using the same convention, with a virtual joint defined between the laboratory global reference frame and the pelvis. In addition to the standard calculation of absolute angles, the offset was calculated by subtracting the corresponding static posture angle from all joint rotations (Leardini et al., 2007).

Total joint range of motion was calculated from peak values (maxima and minima). Then the mean range of motion was calculated from the 20 gait cycles for each joint rotation for each limb. A symmetry index (SI) was calculated using the equation described by Robinson et al. (1987), where:

$$\begin{array}{l} \text{SI}=\{\left(\text{value RJ }-\text{ value LJ}\right)÷[0.5\times (\text{value RJ}\\ \;+\text{value LJ})]\}\times 100 \end{array}$$

In this equation, RJ corresponds to the right joint range of motion value and LJ to the left joint range of motion value. The SI yields a percentage value, which in the case of perfect symmetry is equal to 0%.

### Statistical Analysis

Data are reported as means ± standard deviations. The normality of the distribution of each variable was verified with a Shapiro–Wilk test, and equality of variances was analyzed with a Levene's test.

A Student independent samples *t*-test or Mann–Whitney *U* test was used to compare the baseline data between groups (i.e., G_LLD≤1cm_ vs. G_LLD>1cm_).

A two-way ANOVA with repeated measures was carried out to analyze the effect of the OI on each variable as a function of the group, with the orthosis condition (with/without OI) as the within-subjects factor and the group (G_LLD≤1cm_ vs. G_LLD>1cm_) as the between-subjects factor. Separate ANOVAs were carried out for the longer and shorter legs. Sphericity was verified with a Mauchley test, and if it was not met, the significance of the *F*-ratios was adjusted according to the Greenhouse–Geisser procedure or the Huyn–Feldt procedure. When significant differences were obtained, a Bonferroni *post-hoc* test was conducted to determine where the differences lay.

Statistical significance was set at *p* < 0.05, and all analyses were performed with Statistica software (version 10.0, Statsoft®, Tulsa, OK, United States).

## Results

A total of 46 subjects with anatomic LLD were included in the study (Table 1). Sixteen subjects had an LLD ≤ 1.0 cm, and 30 had an LLD between 1.0 and 3.0 cm. In 14 cases, the shorter leg was on the left, and in 32 cases, it was on the right. There were no significant between-group differences for sex (*p* = 0.536), age (*p* = 0.585), body mass (*p* = 0.775), height (*p* = 0.787), body mass index (*p* = 0.512), or pain rating (*p* = 0.768; Table 1). All the average normalized kinematic curves for each group (G_LLD≤1cm_ and G_LLD>1cm_) without and with orthotic insoles during the gait cycle have been added in the Supplementary Material.

**Table 1:** Sociodemographic, anthropometric characteristics, and pain ratings of the subjects included in each group.

	**G_LLD≤1cm_**	**G_LLD>1cm_**
	***n* = 16**	***n* = 30**
Men (%)	43.8%	53.3%
Age (years)	33.4 ± 12.1	35.5 ± 12.4
Body mass (kg)	70.4 ± 13.1	71.6 ± 12.2
Height (m)	1.74 ± 0.10	1.73 ± 0.09
Body mass index (kg m^−2^)	23.2 ± 3.0	23.8 ± 2.7
Leg length discrepancy (mm)	8.3 ± 1.4	15.1 ± 4.0^a^

a *Significant difference between groups (p < 0.001)*.

### Stance Phase

There was a significant effect of the OI on the SI, with an improvement in the symmetry of pelvic motion in the frontal plane (*p* = 0.001) and ankle motion in the sagittal plane (*p* = 0.010; Table 2). Although, there was a significant effect of the OI on the hip SI in the frontal plane according to the ANOVA (*p* = 0.027), the Bonferroni *post-hoc* test did not show any significant differences between the conditions (*p* = 0.067).

**Table 2:** Symmetry index (with joint range of motion) between the longer and shorter legs for the sagittal, frontal, and transverse planes for the pelvis, hip, knee, and ankle with or without orthotic insoles (OIs) in both groups during the stance and swing phases.

**Phase**	**Joint**	**Plane**	**Without OI**		**With OI**		**Orthosis effect (*p* value)**	**Group effect (*p-*value)**	**Combined effect (*p-*value)**
			**G_LLD≤1cm_**	**G_LLD>1cm_**	**G_LLD≤1cm_**	**G_LLD>1cm_**			
Stance phase	Pelvis	Sagittal (anterior/posterior tilt)	16.5 ± 14.1	19.9 ± 16.3	15.2 ± 15.2	20.0 ± 16.6	0.803	0.340	0.786
		Frontal (upward/downward tilt)	10.8 ± 8.0	14.4 ± 8.2	7.1 ± 6.0	11.2 ± 7.5	0.001^*^	0.085	0.780
		Transverse (internal/external rotation)	5.6 ± 6.1	8.1 ± 7.7	5.6 ± 4.2	8.3 ± 13.3	0.974	0.296	0.949
	Hip	Sagittal (flexion/extension)	4.4 ± 3.6	3.9 ± 2.8	4.9 ± 3.4	3.8 ± 3.1	0.552	0.408	0.345
		Frontal (adduction/abduction)	15.4 ± 9.2	13.4 ± 9.2	10.6 ± 8.1	12.7 ± 10.5	0.027^*^	0.992	0.105
		Transverse (internal/external rotation)	25.6 ± 23.5	33.6 ± 25.8	24.6 ± 23.3	29.6 ± 23.9	0.434	0.344	0.633
	Knee	Sagittal (flexion/extension)	8.8 ± 6.1	11.1 ± 8.3	9.8 ± 6.8	11.4 ± 7.1	0.535	0.334	0.718
		Frontal (adduction/abduction)	24.1 ± 13.7	26.5 ± 20.2	22.3 ± 16.9	30.6 ± 22.1	0.699	0.302	0.345
		Transverse (internal/external rotation)	29.2 ± 24.9	27.0 ± 18.0	24.3 ± 24.1	25.6 ± 18.2	0.270	0.936	0.538
	Ankle	Sagittal (dorsiflexion/plantar flexion)	17.3 ± 7.3	18.1 ± 11.5	13.6 ± 9.5	15.2 ± 10.8	0.010^*^	0.688	0.719
		Frontal (inversion/eversion)	24.8 ± 16.5	25.4 ± 17.4	26.0 ± 19.1	24.6 ± 16.5	0.934	0.941	0.619
		Transverse (internal/external rotation)	36.8 ± 25.5	30.9 ± 22.2	28.8 ± 22.1	27.8 ± 19.7	0.115	0.564	0.487
Swing phase	Pelvis	Sagittal (anterior/postrior tilt)	27.2 ± 14.8	30.4 ± 24.7	35.7 ± 24.0	29.0 ± 25.2	0.320	0.781	0.168
		Frontal (upward/downward tilt)	19.3 ± 12.5	16.9 ± 10.3	17.4 ± 10.5	13.9 ± 9.7	0.054	0.342	0.646
		Transverse (internal/external rotation)	9.7 ± 7.4	11.1 ± 9.3	10.9 ± 9.0	14.6 ± 19.1	0.248	0.475	0.574
	Hip	Sagittal (flexion/extension)	5.0 ± 3.5	5.0 ± 4.6	5.7 ± 4.5	5.0 ± 3.9	0.469	0.757	0.531
		Frontal (adduction/abduction)	21.4 ± 22.7	22.1 ± 20.4	17.5 ± 17.0	22.2 ± 17.4	0.334	0.634	0.309
		Transverse (internal/external rotation)	34.9 ± 26.4	41.2 ± 30.7	37.3 ± 34.4	37.4 ± 24.8	0.864	0.695	0.419
	Knee	Sagittal (flexion/extension)	3.0 ± 1.6	3.7 ± 2.9	3.2 ± 1.6	3.3 ± 2.7	0.778	0.554	0.351
		Frontal (adduction/abduction)	30.9 ± 23.4	42.1 ± 27.6	31.9 ± 18.9	47.4 ± 35.3	0.509	0.083	0.649
		Transverse (internal/external rotation)	30.3 ± 27.0	29.5 ± 22.5	26.7 ± 21.6	26.6 ± 18.3	0.430	0.934	0.924
	Ankle	Sagittal (dorsiflexion/plantar flexion)	14.4 ± 9.5	15.4 ± 13.4	13.2 ± 9.5	15.5 ± 15.4	0.737	0.657	0.668
		Frontal (inversion/eversion)	33.1 ± 19.9	40.8 ± 31.0	28.4 ± 18.6	35.7 ± 31.1	0.294	0.299	0.968
		Transverse (internal/external rotation)	33.0 ± 28.0	31.1 ± 28.3	31.8 ± 25.9	25.9 ± 23.1	0.183	0.619	0.414

* *Significant difference (p < 0.05)*.

**Figure 3:**
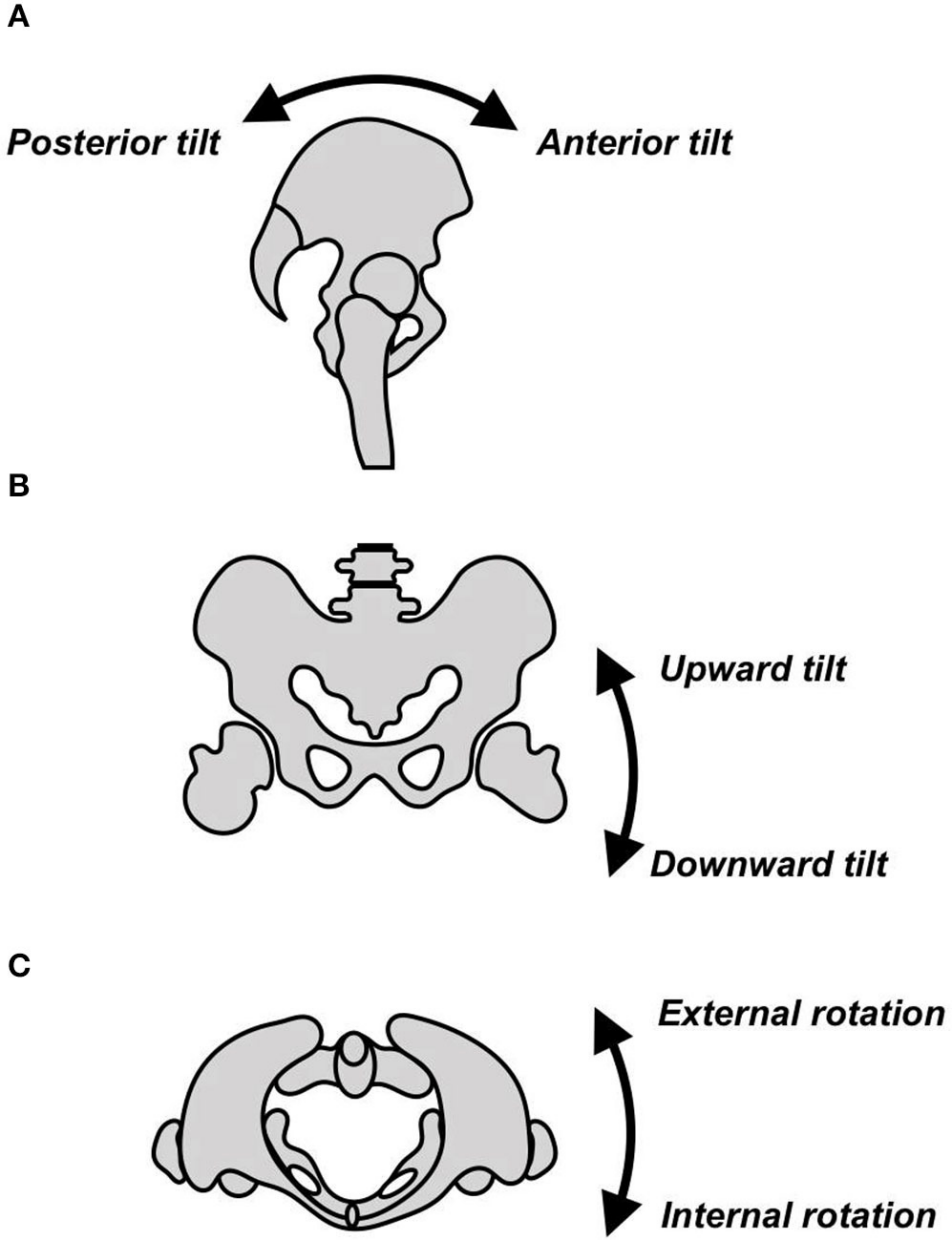
Movement of the pelvis in **(A)** the sagittal plane, **(B)** the frontal plane, and **(C)** the transverse plane.

There was a significant effect of group on the kinematics of several joints. On the side of the longer leg, peak hip adduction was greater (*p* = 0.041), and peak downward lateral pelvic tilt (*p* = 0.035) and peak hip abduction were lower (*p* = 0.044) in the G_LLD>1cm_ (Table 3). On the side of the shorter leg, peak upward pelvic tilt (Figure 3) was greater (*p* = 0.011), and peak downward lateral pelvic tilt was lower (*p* = 0.012) in the G_LLD≤1cm_.

**Table 3:** Peak stance phase angles of the pelvis, hip, knee, and ankle joints for each leg with and without orthotic insoles (OIs) for both groups.

			**Without OI**		**With OI**		**Orthosis effect (*p*-value)**	**Group effect (*p-*value)**	**Combined effect (*p*-value)**
			**G_LLD≤1cm_**	**G_LLD>1cm_**	**G_LLD≤1cm_**	**G_LLD>1cm_**			
Pelvis	Anterior tilt peak	Long	10.2 ± 4.0	10.8 ± 5.7	10.7 ± 3.5	10.9 ± 6.0	0.202	0.803	0.403
		Short	10.2 ± 4.0	10.8 ± 5.6	10.7 ± 3.5	10.9 ± 6.0	0.226	0.798	0.302
	Upward pelvic tilt peak	Long	4.6 ± 1.9	5.8 ± 2.3	4.8 ± 1.8	5.9 ± 2.3	0.417	0.083	0.580
		Short	4.1 ± 1.5	2.5 ± 1.9	4.1 ± 1.7	2.8 ± 2.0	0.021^*^	0.011^*^	0.044^*^
	Internal rotation peak	Long	5.4 ± 2.8	3.7 ± 2.6	5.2 ± 2.7	3.8 ± 2.6	0.716	0.058	0.398
		Short	5.7 ± 2.3	5.5 ± 2.3	6.0 ± 2.5	5.1 ± 2.2	0.820	0.477	0.011^*^
	Posterior tilt peak	Long	7.4 ± 4.5	8.6 ± 5.9	8.1 ± 3.9	8.6 ± 6.2	0.239	0.615	0.203
		Short	7.5 ± 4.4	8.5 ± 5.8	8.2 ± 3.9	8.4 ± 6.1	0.182	0.726	0.122
	Downward pelvic tilt peak	Long	−1.3 ± 1.4	−0.1 ± 1.8	−1.5 ± 1.6	−0.5 ± 1.7	0.003^*^	0.035^*^	0.316
		Short	−2.3 ± 1.7	−3.9 ± 1.9	−2.3 ± 1.5	−3.7 ± 2.0	0.267	0.012^*^	0.213
	External rotation peak	Long	−5.3 ± 2.7	−5.2 ± 2.4	−5.6 ± 3.0	−4.8 ± 2.3	0.692	0.592	0.041^*^
		Short	−5.0 ± 3.1	−3.7 ± 2.6	−4.9 ± 3.1	−3.7 ± 2.7	0.686	0.14	0.627
Hip	Flexion peak	Long	29.4 ± 4.8	31.5 ± 8.1	30.2 ± 4.5	31.5 ± 8.1	0.325	0.434	0.359
		Short	28.8 ± 5.5	30.5 ± 7.9	30.1 ± 5.1	30.9 ± 8	0.013^*^	0.576	0.143
	Extension peak	Long	−8.8 ± 4.8	−8.5 ± 7.0	−8.5 ± 4.9	−8.8 ± 7.3	0.985	0.993	0.48
		Short	−9.7 ± 5.2	−9.9 ± 7.3	−9.1 ± 5.5	−10.1 ± 7.7	0.496	0.795	0.324
	Adduction peak	Long	7.7 ± 3.5	9.9 ± 2.9	7.9 ± 3.8	9.8 ± 2.7	0.612	0.041^*^	0.373
		Short	7.2 ± 2.8	5.8 ± 3.5	7.2 ± 2.8	6.1 ± 3.7	0.128	0.221	0.114
	Abduction peak	Long	−0.7 ± 3.3	1.3 ± 2.6	−0.6 ± 3.4	1.0 ± 2.5	0.366	0.044^*^	0.08
		Short	−2.3 ± 3.0	−3.0 ± 3.7	−2.1 ± 2.5	−2.6 ± 3.9	0.046^*^	0.578	0.343
	Internal rotation peak	Long	5.6 ± 7.9	5.2 ± 7.5	7.3 ± 8.2	4.9 ± 7.6	0.297	0.554	0.136
		Short	6.2 ± 9.4	4.7 ± 7.4	6.0 ± 8.2	4.2 ± 7.7	0.600	0.482	0.829
	External rotation peak	Long	−2.6 ± 7.0	−3.3 ± 7.4	−1.5 ± 6.4	−3.8 ± 7.4	0.644	0.491	0.186
		Short	−2.2 ± 8.2	−5.1 ± 7.7	−2.8 ± 6.7	−5.7 ± 7.5	0.409	0.200	0.911
Knee	Flexion peak	Long	24.8 ± 4.6	23.2 ± 4.5	25.4 ± 5.1	23.5 ± 5.2	0.102	0.247	0.558
		Short	24.2 ± 3.9	22.6 ± 3.9	24.3 ± 3.5	22.5 ± 4.6	0.997	0.164	0.728
	Extension peak	Long	−3.8 ± 3.3	−2.6 ± 4.2	−4.1 ± 3.7	−2.7 ± 4.1	0.434	0.288	0.630
		Short	−4.5 ± 2.7	−3.7 ± 3.6	−4.5 ± 3.6	−3.5 ± 3.7	0.636	0.402	0.772
	Adduction peak	Long	2.9 ± 3.9	2.6 ± 3.7	3.3 ± 4.5	2.6 ± 3.5	0.325	0.671	0.424
		Short	2.7 ± 3.7	2.4 ± 3.9	2.8 ± 3.7	2.3 ± 4.0	0.989	0.699	0.655
	Abduction peak	Long	−1.9 ± 3.6	−2.4 ± 3.6	−1.9 ± 3.8	−2.5 ± 3.2	0.639	0.606	0.744
		Short	−2.5 ± 3.4	−2.2 ± 3.5	−1.8 ± 3.7	−2.8 ± 3.8	0.854	0.066	0.006^*^
	Internal rotation peak	Long	−12.1 ± 7.3	−10.4 ± 9.9	−14.0 ± 7.3	−10.3 ± 9.8	0.196	0.315	0.170
		Short	−10.4 ± 8.4	−8.9 ± 7.2	−9.6 ± 7.1	−9.2 ± 8.0	0.745	0.659	0.531
	External rotation peak	Long	−25.3 ± 8.5	−23.7 ± 9.9	−27.4 ± 10.2	−23.3 ± 9.8	0.221	0.335	0.097
		Short	−25.1 ± 9.3	−21.8 ± 8.7	−23.5 ± 8.4	−22.0 ± 9.8	0.442	0.370	0.316
Ankle	Dorsiflexion peak	Long	14.4 ± 4.1	14.7 ± 2.9	13.8 ± 4.0	14.4 ± 3.2	0.011^*^	0.708	0.449
		Short	11.3 ± 4.6	12.0 ± 3.6	11.5 ± 4.5	12.6 ± 3.5	0.028^*^	0.457	0.277
	Plantar flexion peak	Long	−7.3 ± 4.4	−6.6 ± 2.4	−7.0 ± 4.4	−6.1 ± 2.5	0.012^*^	0.416	0.707
		Short	−7.5 ± 4.1	−6.8 ± 3.3	−7.2 ± 4.3	−6.5 ± 3.6	0.187	0.525	0.892
	Inversion peak	Long	12.4 ± 3.7	12 ± 3.5	13.1 ± 3.6	12.3 ± 3.4	0.018^*^	0.570	0.365
		Short	14.7 ± 4.8	12.5 ± 3.8	14.7 ± 4.1	12.7 ± 3.7	0.712	0.092	0.760
	Eversion peak	Long	2.6 ± 3.3	2.0 ± 3.1	3.0 ± 3.3	2.9 ± 2.8	0.001^*^	0.737	0.193
		Short	3.3 ± 3.2	3.2 ± 3.3	3.2 ± 3.7	3.7 ± 3.2	0.337	0.854	0.168
	Internal rotation peak	Long	−2.9 ± 6.5	−6.8 ± 4.9	−3.4 ± 7.0	−6.5 ± 5.9	0.700	0.054	0.328
		Short	−3.9 ± 4.2	−4.0 ± 5.2	−3.5 ± 4.1	−4.7 ± 5.2	0.542	0.641	0.023^*^
	External rotation peak	Long	−8.5 ± 6.5	−11.4 ± 5.1	−8.9 ± 6.4	−11.3 ± 5.9	0.627	0.150	0.455
		Short	−9.6 ± 3.7	−9.1 ± 5.3	−9.2 ± 3.3	−9.5 ± 4.9	0.854	0.938	0.084

* *Significant difference (p < 0.05)*.

There was a significant effect of OI on the kinematics of several joints. On the side of the longer leg, peak downward lateral pelvic tilt (*p* = 0.003) and peak ankle inversion (*p* = 0.018) were significantly increased with the OI compared to without the OI. Peak ankle eversion (*p* = 0.001), peak ankle dorsiflexion (*p* = 0.011), and peak ankle plantarflexion (*p* = 0.012) were significantly decreased with the OI (Table 3). On the side of the shorter leg, peak upward lateral pelvic tilt angle (*p* = 0.021), peak hip flexion angle (*p* = 0.013), and peak ankle dorsiflexion angle (*p* = 0.028) were significantly increased, and peak hip abduction angle (*p* = 0.046) was significantly decreased with the OI.

Although the ANOVA showed an interaction between OI and group for the shorter leg for peak ankle internal rotation (*p* = 0.023) and peak knee abduction (*p* = 0.006), the Bonferroni *post-hoc* test indicated there were no significant differences between these factors. There was a significant interaction between OI and group for peak upward pelvic tilt on the side of the shorter leg: the Bonferroni *post-hoc* test indicated that without the OI, peak upward pelvic tilt was greater in the G_LLD≤1cm_ (*p* = 0.044) than the G_LLD>1cm_.

There was a significant interaction between OI and group for peak upward pelvic tilt: the Bonferroni *post-hoc* test indicated that with the OI, peak upward pelvic tilt increased significantly in the G_LLD>1cm_ (*p* = 0.04), compared to without the OI.

### Swing Phase

There was a significant effect of group on the SI for several joints. On the side of the longer leg, peak upward pelvic tilt and peak hip adduction angle (*p* = 0.007) were significantly lower in the G_LLD≤1cm_ (*p* = 0.009), and downward lateral pelvic tilt was greater (*p* = 0.013; Table 4). On the side of the shorter leg, peak external pelvic rotation was significantly greater in the G_LLD≤1cm_ (*p* = 0.014).

**Table 4:** Peak swing phase angles of the pelvis, hip, knee, and ankle joints for each leg with and without orthotic insoles (OI) for both groups.

			**Without OI**		**With OI**		**Orthosis effect (*p*-value)**	**Group effect (*p*-value)**	**Combined effect (*p*-value)**
			**G_LLD≤1cm_**	**G_LLD>1cm_**	**G_LLD≤1cm_**	**G_LLD>1cm_**			
Pelvis	Anterior tilt peak	Long	9.9 ± 4.0	10.5 ± 5.6	10.4 ± 3.5	10.5 ± 5.9	0.295	0.826	0.242
		Short	9.8 ± 4.2	10.6 ± 5.7	10.4 ± 3.6	10.6 ± 6.1	0.209	0.767	0.294
	Upward pelvic tilt peak	Long	0.7 ± 1.3	2.1 ± 1.6	0.5 ± 1.5	1.7 ± 1.6	0.001^*^	0.009^*^	0.175
		Short	−0.6 ± 1.7	−1.7 ± 2.0	−0.6 ± 1.7	−1.6 ± 2.0	0.418	0.077	0.654
	Internal rotation peak	Long	4.3 ± 3.0	3.0 ± 2.8	4.3 ± 3.1	3.0 ± 3.0	0.983	0.168	0.87
		Short	4.6 ± 2.7	4.5 ± 2.3	4.8 ± 3.0	4.2 ± 2.3	0.473	0.615	0.101
	Posterior tilt peak	Long	7.5 ± 4.5	8.4 ± 5.7	8.2 ± 4.0	8.4 ± 6.1	0.255	0.758	0.177
		Short	7.4 ± 4.4	8.7 ± 5.9	8.1 ± 3.9	8.7 ± 6.2	0.212	0.579	0.165
	Downward pelvic tilt peak	Long	−4.0 ± 1.5	−2.5 ± 1.9	−4.1 ± 1.7	−2.8 ± 2.0	0.013^*^	0.013^*^	0.127
		Short	−4.6 ± 1.9	−5.8 ± 2.3	−4.7 ± 1.7	−5.9 ± 2.3	0.502	0.073	0.672
	External rotation peak	Long	−5.4 ± 2.3	−5.0 ± 2.3	−5.5 ± 2.4	−4.7 ± 2.2	0.469	0.459	0.173
		Short	−5.1 ± 2.4	−3.2 ± 2.2	−4.9 ± 2.5	−3.2 ± 2.2	0.617	0.014^*^	0.425
Hip	Flexion peak	Long	31.6 ± 5.1	33.7 ± 7.3	31.8 ± 4.9	33.4 ± 7.5	0.890	0.367	0.515
		Short	30.4 ± 5.9	32.1 ± 7.7	31.3 ± 5.2	32.5 ± 8	0.092	0.521	0.435
	Extension peak	Long	−5.8 ± 4.7	−5.7 ± 6.6	−5.2 ± 4.6	−5.8 ± 6.9	0.461	0.897	0.355
		Short	−6.3 ± 5.5	−6.6 ± 7.2	−5.7 ± 5.4	−6.7 ± 7.5	0.567	0.765	0.312
	Adduction peak	Long	1.1 ± 3.3	3.9 ± 2.6	1.2 ± 3.7	3.6 ± 2.5	0.363	0.007^*^	0.163
		Short	−0.1 ± 2.7	−0.6 ± 3.6	0.0 ± 2.3	−0.4 ± 3.8	0.092	0.673	0.34
	Abduction peak	Long	−4.7 ± 3.4	−2.7 ± 2.6	−4.4 ± 3.4	−2.8 ± 2.6	0.720	0.051	0.089
		Short	−5.6 ± 2.6	−6.5 ± 3.4	−5.4 ± 2.1	−6.3 ± 3.5	0.091	0.350	0.582
	Internal rotation peak	Long	5.5 ± 7.4	4.6 ± 7.6	6.7 ± 7.6	4.1 ± 7.5	0.651	0.438	0.179
		Short	5.1 ± 8.9	3.5 ± 7.0	5.1 ± 7.5	2.8 ± 7.4	0.614	0.374	0.609
	External rotation peak	Long	−2.5 ± 6.8	−3.0 ± 7.3	−1.2 ± 6.2	−3.7 ± 7.4	0.603	0.482	0.124
		Short	−2.0 ± 8.3	−4.4 ± 7.6	−2.2 ± 7.0	−5.1 ± 7.3	0.478	0.243	0.691
Knee	Flexion peak	Long	65.0 ± 3.3	65.0 ± 4.4	63.9 ± 3.1	64.4 ± 4.5	0.003^*^	0.834	0.319
		Short	62.7 ± 3.4	63.0 ± 4.2	62.0 ± 3.3	63.0 ± 4.3	0.256	0.594	0.24
	Extension peak	Long	−3.6 ± 3.6	−2.7 ± 4.3	−4.1 ± 4.0	−2.8 ± 4.1	0.193	0.369	0.425
		Short	−4.6 ± 3.4	−3.5 ± 3.9	−4.6 ± 3.7	−3.4 ± 3.9	0.688	0.308	0.644
	Adduction peak	Long	6.7 ± 5.6	6.2 ± 5.5	7.4 ± 6.5	5.7 ± 5.3	0.828	0.505	0.262
		Short	5.8 ± 5.3	5.3 ± 4.9	5.5 ± 4.9	4.6 ± 4.6	0.314	0.635	0.636
	Abduction peak	Long	−2.8 ± 3.7	−2.9 ± 4.1	−2.3 ± 4.3	−2.9 ± 3.6	0.286	0.783	0.249
		Short	−3.7 ± 2.8	−3.0 ± 4.3	−2.9 ± 2.8	−3.4 ± 4.3	0.528	0.913	0.162
	Internal rotation peak	Long	−16.6 ± 6.2	−15.3 ± 9.2	−18.3 ± 7.6	−15.3 ± 8.9	0.218	0.400	0.176
		Short	−15.1 ± 8.2	−12.7 ± 7.1	−14.5 ± 7.9	−13.0 ± 8.2	0.794	0.394	0.587
	External rotation peak	Long	−28.6 ± 9.1	−26.7 ± 9.1	−30.3 ± 10.2	−26.7 ± 8.5	0.233	0.319	0.199
		Short	−26.5 ± 8.9	−24.0 ± 7.8	−25.4 ± 8.3	−24.7 ± 8.1	0.831	0.522	0.217
Ankle	Dorsiflexion peak	Long	3.2 ± 4.9	3.8 ± 3.8	2.7 ± 4.6	4.0 ± 4.2	0.523	0.449	0.207
		Short	0.9 ± 4.2	2.5 ± 3.3	2.0 ± 3.6	3.6 ± 2.9	0.001^*^	0.124	0.886
	Plantar flexion peak	Long	−18.2 ± 7.9	−16.1 ± 5.1	−18.4 ± 7.3	−16.5 ± 5.5	0.367	0.291	0.869
		Short	−21.4 ± 8.4	−18.2 ± 6.1	−20.9 ± 7.8	−17.5 ± 6.2	0.092	0.120	0.706
	Inversion peak	Long	12.4 ± 3.9	11.7 ± 3.4	13.0 ± 4.0	11.8 ± 3.6	0.068	0.390	0.251
		Short	14.8 ± 4.9	12.4 ± 3.8	15.0 ± 4.0	12.7 ± 3.5	0.402	0.056	0.891
	Eversion peak	Long	4.8 ± 3.6	5.4 ± 4.4	5.1 ± 4.1	5.7 ± 4.2	0.324	0.660	0.87
		Short	6.4 ± 4.2	6.2 ± 3.8	6.6 ± 4.5	6.1 ± 3.4	0.896	0.793	0.557
	Internal rotation peak	Long	−2.4 ± 7.9	−6.1 ± 5.1	−3.1 ± 7.7	−6.1 ± 6.1	0.350	0.092	0.360
		Short	−3.0 ± 5.0	−3.2 ± 5.6	−2.9 ± 5.0	−3.7 ± 5.5	0.359	0.768	0.299
	External rotation peak	Long	−14.5 ± 7.5	−16.1 ± 6.0	−15.4 ± 7.5	−16.3 ± 6.8	0.119	0.559	0.336
		Short	−13.1 ± 5.5	−13.3 ± 6.0	−13.2 ± 5.0	−13.6 ± 6.0	0.330	0.877	0.665

* *Significant difference (p < 0.05)*.

There was a significant effect of OI on the kinematics of several joints. On the side of the longer leg, peak upward pelvic tilt (*p* = 0.001), peak knee flexion decreased (*p* = 0.003), and peak downward pelvic tilt (*p* = 0.013) increased significantly with the OI (Table 4).

On the side of the shorter leg, there was a significant increase in peak ankle dorsiflexion (*p* < 0.001).

### Pain

There was a significant effect of OI on pain (*p* < 0.001). The pain reduced from 5.9 ± 1.8 to 1.7 ± 2.1 in G_LLD≤1cm_ and from 5.7 ± 2.6 to 2.0 ± 2.5 in G_LLD>1cm_. There was no group effect (*p* = 0.929) (Table 5).

**Table 5:** Localization of pain of the subjects included in each group.

	**G_LLD≤1cm_**		**G_LLD>1cm_**	
	***n*** **= 16**		***n*** **= 30**	
**Low back pain**	9		17	
**Hip pain**	4		3	
**Knee pain**	3		4	
**Ankle pain**	0		6	
	**G_LLD≤1cm_**		**G_LLD>1cm_**	
	**Without OI**	**With OI**	**Without OI**	**With OI**
**Visual analog scale scores**	5.9 ± 1.8	1.7 ± 2.1	5.7 ± 2.6	2.0 ± 2.5

## Discussion

The aim of this study was to evaluate the immediate effect of OI on gait kinematics and pain in subjects with mild LLD according to two groups of the leg length discrepancy.

The results of this study demonstrated that gait symmetry improved with the OI, particularly at the pelvis (frontal plane) and ankle (sagittal plane) during the stance phase of gait, with no between-group differences. Moreover, there was a significant reduction in pain with the OI (with no between-group differences). The kinematic results support the findings of a number of studies that showed that even mild LLD can alter the kinematics of gait and cause pain (Perttunen et al., 2004; Defrin et al., 2005; Golightly et al., 2007; Seeley et al., 2010; Khamis and Carmeli, 2018). The results of this study add to this body of knowledge by showing that even LLD <1 cm can alter symmetry and cause pain, and that both symmetry and pain can be improved with OI.

The results of this study confirm previous findings that deviations of pelvic motion in the frontal plane are common in LLD (Giles, 1981; Giles and Taylor, 1982; Walsh et al., 2000; Golightly et al., 2007; Jamaluddin et al., 2011; Resende et al., 2016). There was a significant increase in peak pelvic downward lateral tilt on the side of the longer leg with the OI, and a concomitant increase in peak upward lateral tilt on the side of the shorter leg (*p* = 0.021) in both groups with the OI. Similar results have previously been found with the use of OI in subjects with moderate and severe LLD (Bangerter et al., 2019). The present results showed that OIs have a similar effect in mild LLD ≤ 1 and >1 cm. The increase in ankle dorsiflexion on the longer leg (Walsh et al., 2000; Resende et al., 2016) and the increase in the plantar flexion on the shorter leg during stance phase are also in line with the results of previous studies (Song et al., 1997; Walsh et al., 2000; Aiona et al., 2015; Resende et al., 2016). The results of the present study showed that use of an OI significantly increased peak dorsiflexion in the shorter leg and decreased both peak dorsiflexion and peak plantar flexion in the longer leg (independently from the group). These changes likely contributed to the improvement in ankle gait symmetry shown by the SI. The kinematic alterations found at the pelvis (frontal plane) and ankle (sagittal plane) during gait without the OI are typical compensatory strategies that functionally lengthen the shorter limb and shorten the longer limb (Resende et al., 2016). The findings of the present study indicate that the OI reduced the need for such strategies.

The results of several studies in the literature contrast with those of the present study: some studies found no effect of an OI on joint kinematics during gait in mild LLD (Bandy and Sinning, 1986; Goel et al., 1997), although effects were found for moderate and severe LLD (Bangerter et al., 2019). These different results could be due to differences in the study methodologies. First, the sample sizes in both the studies by Bandy and Sinning (1986) and Goel et al. (1997) were smaller than that of the present study, and they may have been underpowered. The studies also analyzed different variables and used different types of LLD correction: Bandy and Sinning (1986) used a heel lift, while Goel et al. (1997) used a shoe lift. Although Bandy and Sinning (1986) found that the heel lift seemed to bring about more symmetrical movement, another study (Khamis and Carmeli, 2017) found that a heel lift was insufficient to affect the entire stance phase of the gait cycle.

The positive effect of the OI on symmetry found in the present study for both mild and very mild LLD was further supported by the significant reduction in pain: use of the OI immediately and significantly reduced pain in both groups, with no between-group differences. These results are clinically important since the biomechanical, postural, and functional changes caused by LLD have been shown to alter joint angles (Gurney, 2002; Campbell et al., 2018), leading to low back pain, scoliosis, pelvic and sacral misalignments, hip and knee osteoarthritis, and even stress fractures of the lower limbs (Gurney, 2002; Kendall et al., 2014; Campbell et al., 2018; Beeck et al., 2019). The reduction of pelvic obliquity with the OI likely reduced muscle overactivity (Mannello, 1992) and the distribution forces on the spinal joints, thus reducing pain (Defrin et al., 2005; Golightly et al., 2007) and potentially reducing the development of pathology in the long-term (Giles, 1981; Giles and Taylor, 1982; Cummings et al., 1993). LLD has been implicated in hip and knee pain due to inadequate distribution of mechanical loads (McCaw and Bates, 1991; McWilliams et al., 2013). Indeed, LLD results in excessive and uneven loading on the hip and/or knee and also on the mobile segments of the lumbar belt (Murray and Azari, 2015). Improvement of the gait symmetry of the pelvis in the frontal plane and of the ankle in the sagittal plane could improve the distribution of mechanical loads throughout the lower limb and thus significantly reduce associated pain. These results are in line with the current literature, which shows that OI can reduce pain in subjects with mild LLD (Defrin et al., 2005; Golightly et al., 2007; Menez et al., 2020). In addition, as reported by Defrin et al. (2005) who evaluated only the effect of insoles on low back pain, very mild LLD can be the source of pain, and the shoe inserts can be a suitable therapeutic solution to reduce pain. Longitudinal studies are now required to determine the long-term effect of OI on chronic pain.

As found in a previous study (Resende et al., 2016), peak hip flexion was increased, and peak hip abduction was decreased on the side of the shorter leg during stance without the OI. Although these deviations were reduced with the OI, the SI did not change for these joints in either group, suggesting that the use of the OI was insufficient to correct them. This was also the case for ankle inversion–eversion during stance on the side of the longer leg, as well as the deviations found in pelvic, knee, and ankle motion during swing phase (Table 4). Several factors could explain the lack of normalization of these kinematic parameters with the OI. First, it is possible that the trim magnitude of the OI was too low (the correction applied was 50% of the magnitude of the LLD). Clinically, it may be worthwhile to carry out repeated kinematic analyses with OI of different magnitudes until all joint kinematics become symmetrical left–right. Second, and more likely, the compensatory strategies for the LLD were well-established in these individuals with anatomical LLD, and it is thus unsurprising that their strategies could not be changed in a single session of walking with OI. Moreover, the compensatory biomechanical strategies used by subjects with LLD are complex (Menez et al., 2020). Further studies are required to assess the longer-term effects of OI on gait kinematics.

The present study adds to the current body of literature on LLD by providing more extensive kinematic data. Together, these results confirm that even mild LLD alters gait kinematics.

However, new studies are essential to continue to optimize the management of subjects in the field of podiatry. These future studies will need to consider some of the limitations identified throughout this work. This study was not a randomized controlled trial, and neither the examiner nor the participants were blinded that can lead to a placebo effect of OI for pain assessment. For pain analysis, we have adapted to the field conditions using a visual analog scale. In the clinical and research field, the visual analog scale is widely used and accepted (Hayashi et al., 2015). We have tried to limit the potential bias that comes with the subjective declaration of the visual analog scale by asking subjects to be as truthful as possible in their evaluations. Future trials should be blinded to reduce this potential bias. On the other hand, more precise questionnaires should be implemented in order to situate and define pain more accurately, as some previous studies have done (Defrin et al., 2005; Golightly et al., 2007). For the SI, we can observe (Table 2) that some SIs are higher for the G_LLD≤1cm_ than for the G_LLD>1cm_ (especially in the transverse plane). A limitation of the SI is the potential for artificial inflation. This inflation can occur when the observed variables have small changes that can lead to large changes in the SI (Cabral et al., 2016). Finally, it would be interesting to highlight other aspects of motion analysis that could complete and explain some of our results. Indeed, with a kinetic approach, Aiona et al. (2015) and Song et al. (1997) put forward a more important mechanical work of the long leg, therefore possibly a more important articular, muscular, and tendinous work, which was confirmed by Perttunen et al. (2004). In future studies, it would be interesting to supplement the kinematic data with kinetic variables coupled with electromyographic analysis to refine the understanding of the effect of OI on changes in the biomechanics of locomotion.

## Conclusion

This study contributes to a better understanding of the effect of OI on gait kinematics observed in subjects with mild LLD. OI immediately significantly improved the articular symmetry of the pelvis in the frontal plane and of the ankle in the sagittal plane, regardless of the height of LLD (i.e., LLD ≤ 1 cm vs. LLD > 1 cm <3 cm). In addition, our study confirms that OI significantly reduces pain in subjects with mild LLD. Therefore, we can recommend treatment of mild LLD with OI, even when LLD ≤ 1 cm. This study contributes to a better understanding of the effect of OI on gait kinematics in subjects with mild LLD and provides valuable information for clinicians. Nevertheless, future studies could complement this research and shed new light on this research subject.

## Data Availability Statement

The raw data supporting the conclusions of this article will be made available by the authors, without undue reservation.

## Ethics Statement

Ethical review and approval was not required for the study on human participants in accordance with the local legislation and institutional requirements. The patients/participants provided their written informed consent to participate in this study.

## Author Contributions

All of the named authors meet the criteria for authorship. CM was the principal investigator. He co-designed the study, over-saw the project, contributed to the interpretation of the data, revised the study report for intellectual content, and approved the version to be published. JC co-designed the study, contributed to the interpretation of the data, coordinate the project, contributed to the drafting of the manuscript, revised it for intellectual content, and approved the version to be published. ML'H co-designed the study, coordinated the project, revised the report for intellectual content, and approved the version to be published.

## Conflict of Interest

The authors declare that the research was conducted in the absence of any commercial or financial relationships that could be construed as a potential conflict of interest.

## Abbreviations

G_LLD≤1cm_: Group Leg Length Discrepancy ≤ 1 cm
G_LLD>1cm_: Group Leg Length Discrepancy > 1 cm
LLD: Leg Length Discrepancy
OI: Orthotic Insoles
SI: Symmetry Index
STROBE: STrengthening the Reporting of OBservational studies in Epidemiology.
