# Supplementary material for: Orthotic Insoles Improve Gait Symmetry and Reduce Immediate Pain in Subjects With Mild Leg Length Discrepancy
Source: Front Sports Act Living. 2020 Dec 16;2:579152. doi: 10.3389/fspor.2020.579152 (PMC7750876; doi:10.3389/fspor.2020.579152)
Supplement: Supplementary file 1 [file Data_Sheet_1.PDF]

Supplementary material

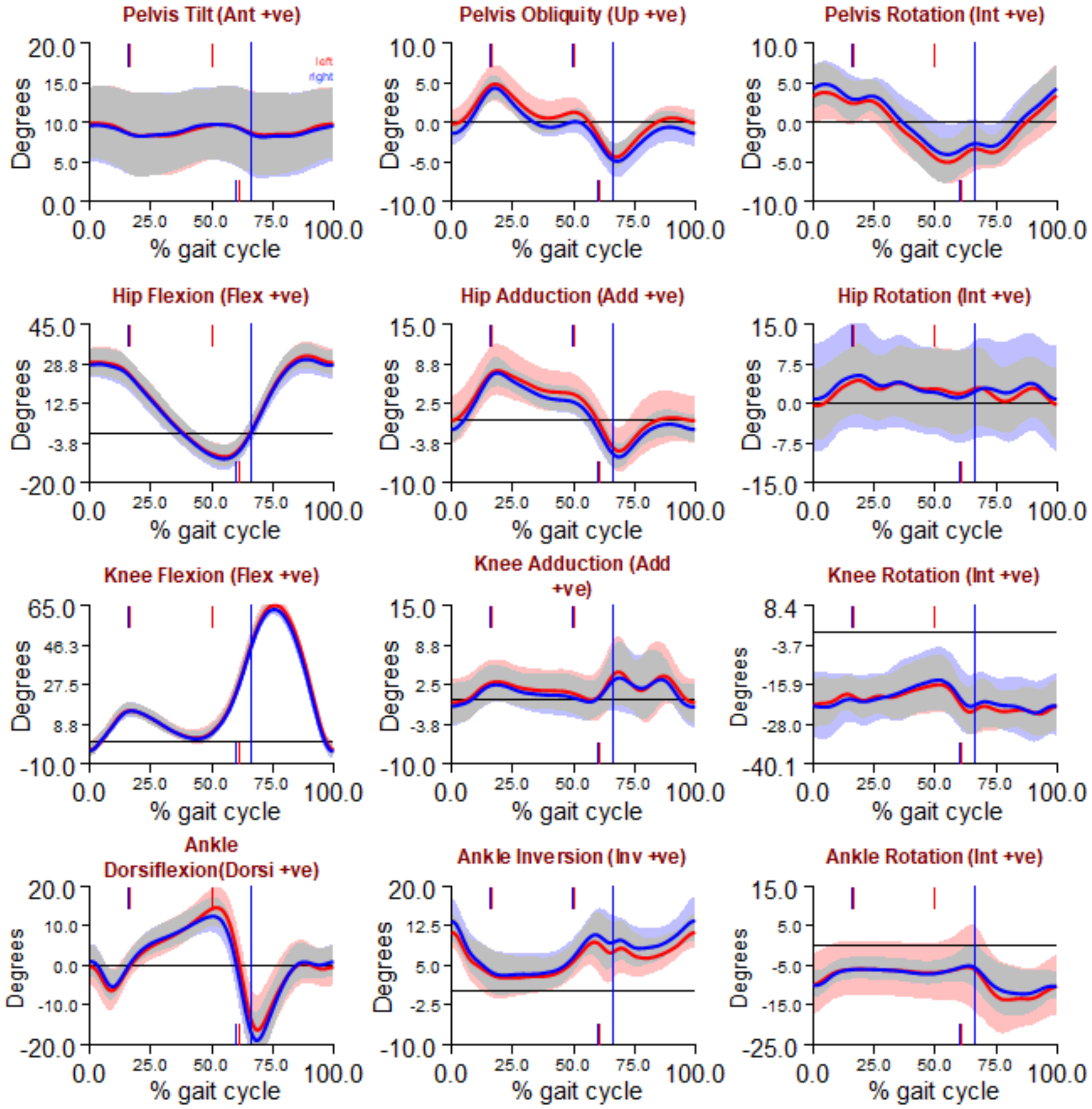

Figure 1S. Average normalized kinematic curves for group with leg length discrepancy ≤1cm without orthotic insoles during the gait cycle. The blue curves represent the data for the right leg (short leg) and the red curves the data for the left leg (long leg). Between-curve standard deviation is shaded in blue for the right leg and in red for the left leg. The stance phase and the swing phase are separated by the vertical line in blue.

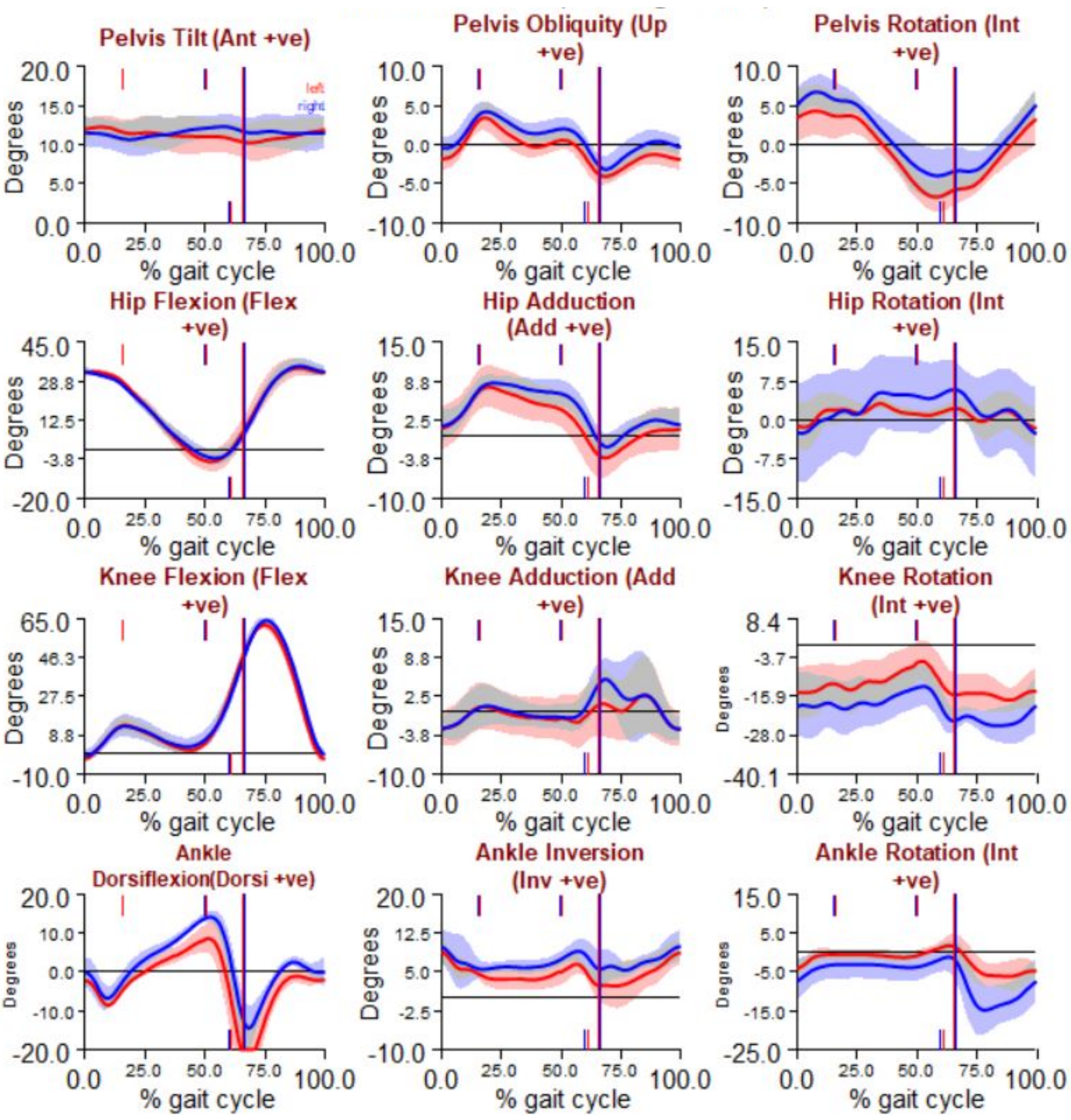

Figure 2S. Average normalized kinematic curves for group with leg length discrepancy ≤1cm without orthotic insoles during the gait cycle. The blue curves represent the data for the right leg (long leg) and the red curves the data for the left leg (short leg). Between-curve standard deviation is shaded in blue for the right leg and in red for the left leg. The stance phase and the swing phase are separated by the vertical line in blue.

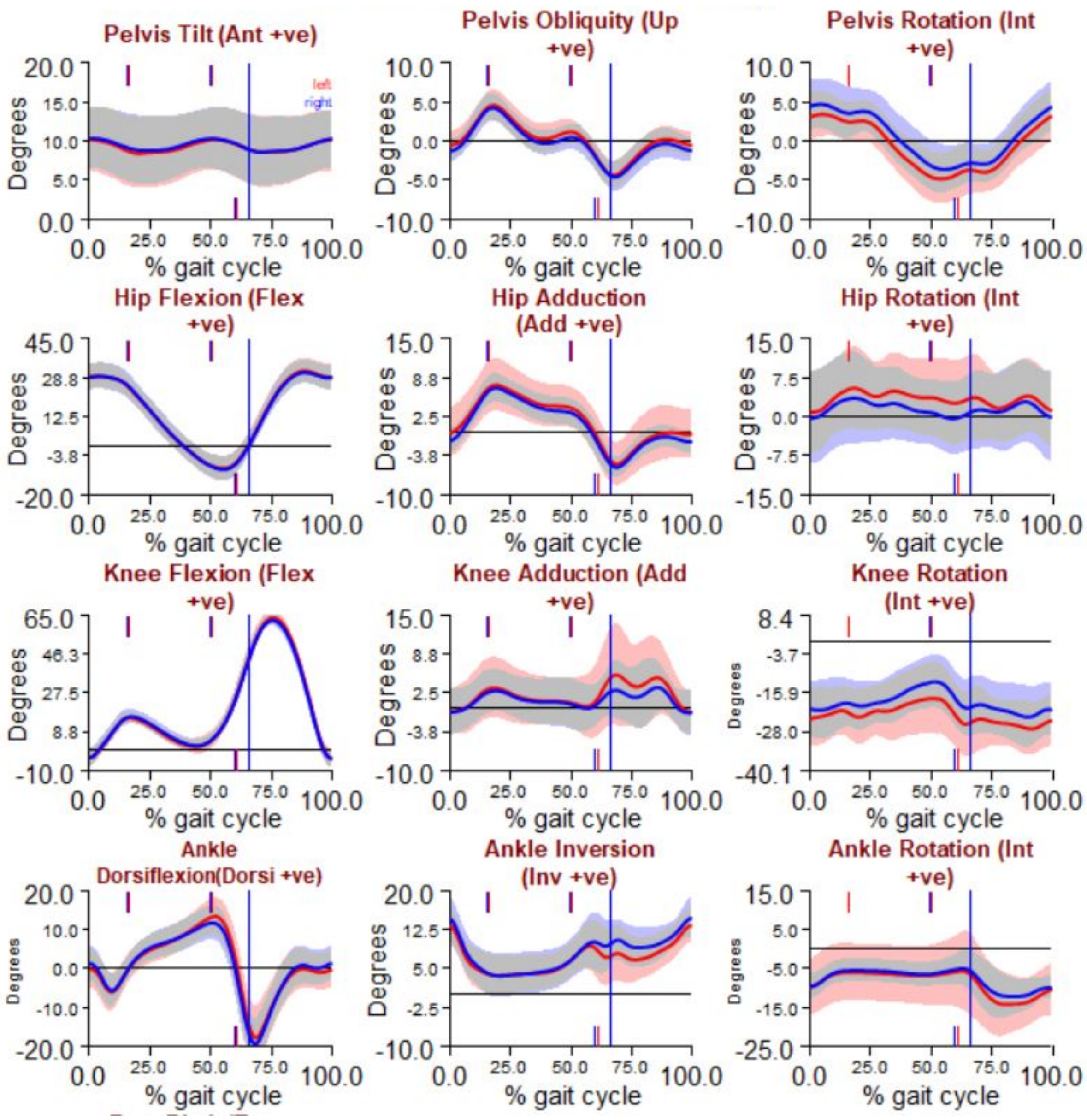

Figure 3S. Average normalized kinematic curves for group with leg length discrepancy ≤1cm with orthotic insoles during the gait cycle. The blue curves represent the data for the right leg (short leg) and the red curves the data for the left leg (long leg). Between-curve standard deviation is shaded in blue for the right leg and in red for the left leg. The stance phase and the swing phase are separated by the vertical line in blue.

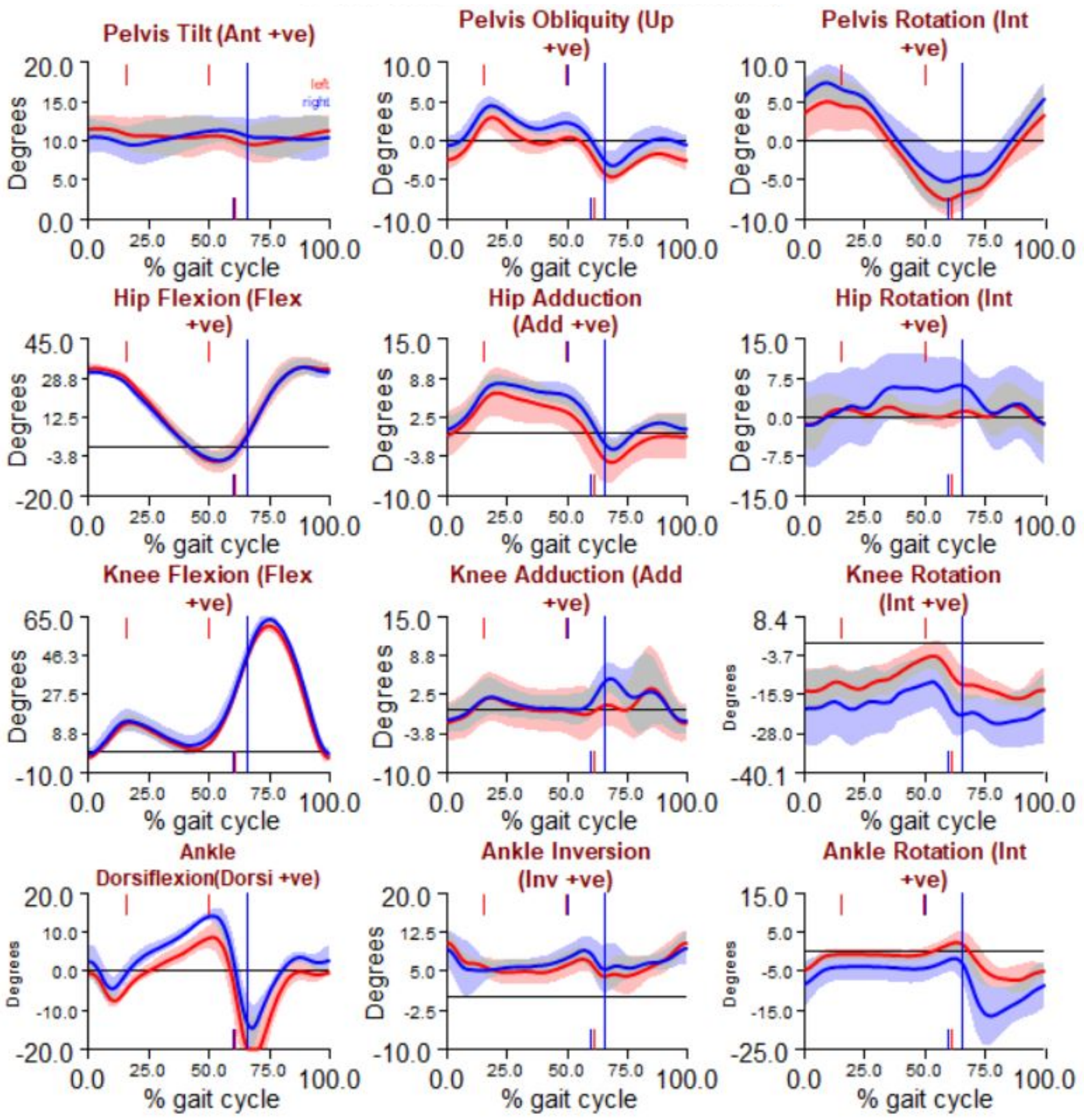

Figure 4S. Average normalized kinematic curves for group with leg length discrepancy ≤1cm with orthotic insoles during the gait cycle. The blue curves represent the data for the right leg (long leg) and the red curves the data for the left leg (short leg). Between-curve standard deviation is shaded in blue for the right leg and in red for the left leg. The stance phase and the swing phase are separated by the vertical line in blue.

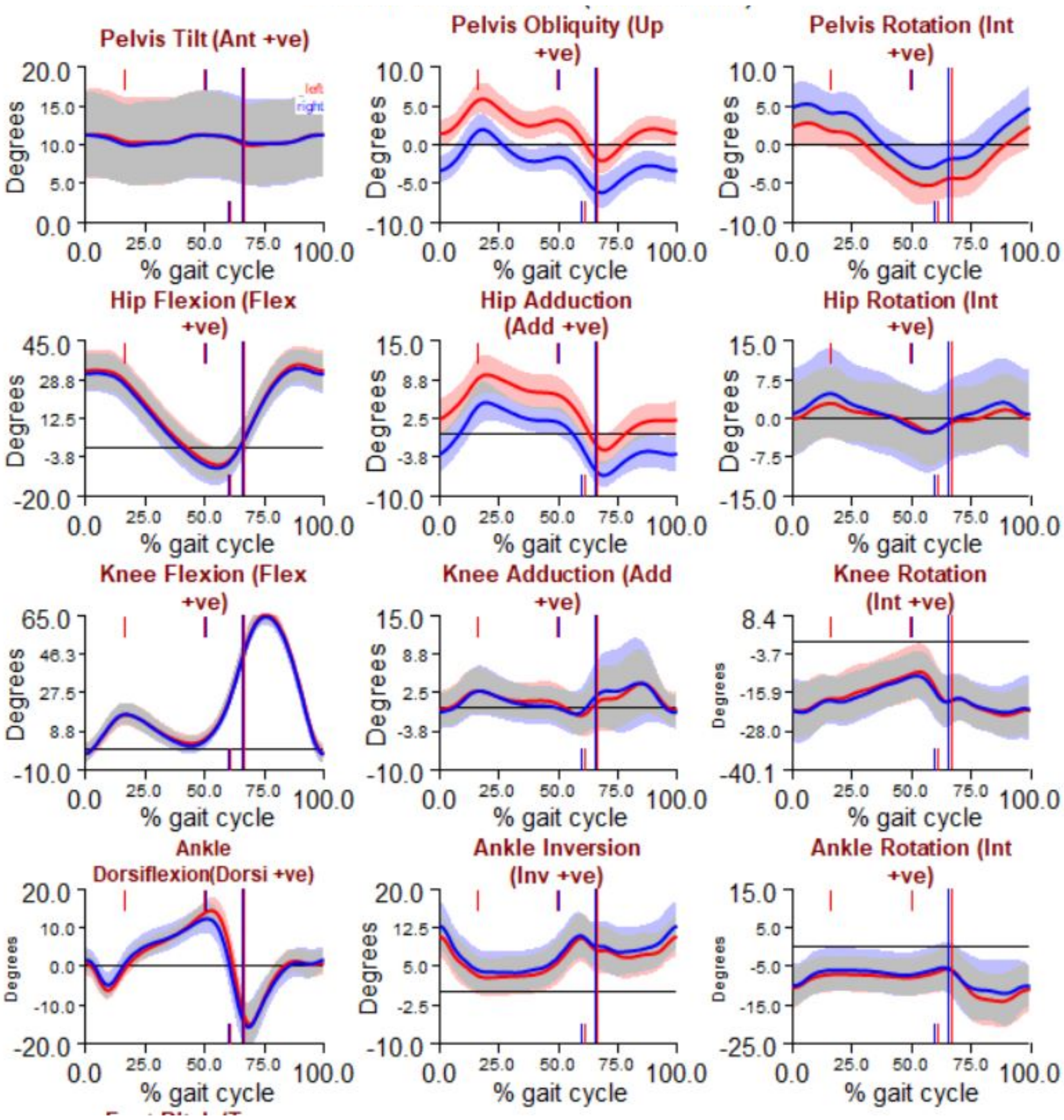

Figure 5S. Average normalized kinematic curves for group with leg length discrepancy >1cm without orthotic insoles during the gait cycle. The blue curves represent the data for the right leg (short leg) and the red curves the data for the left leg (long leg). Between-curve standard deviation is shaded in blue for the right leg and in red for the left leg. The stance phase and the swing phase are separated by the vertical line in blue.

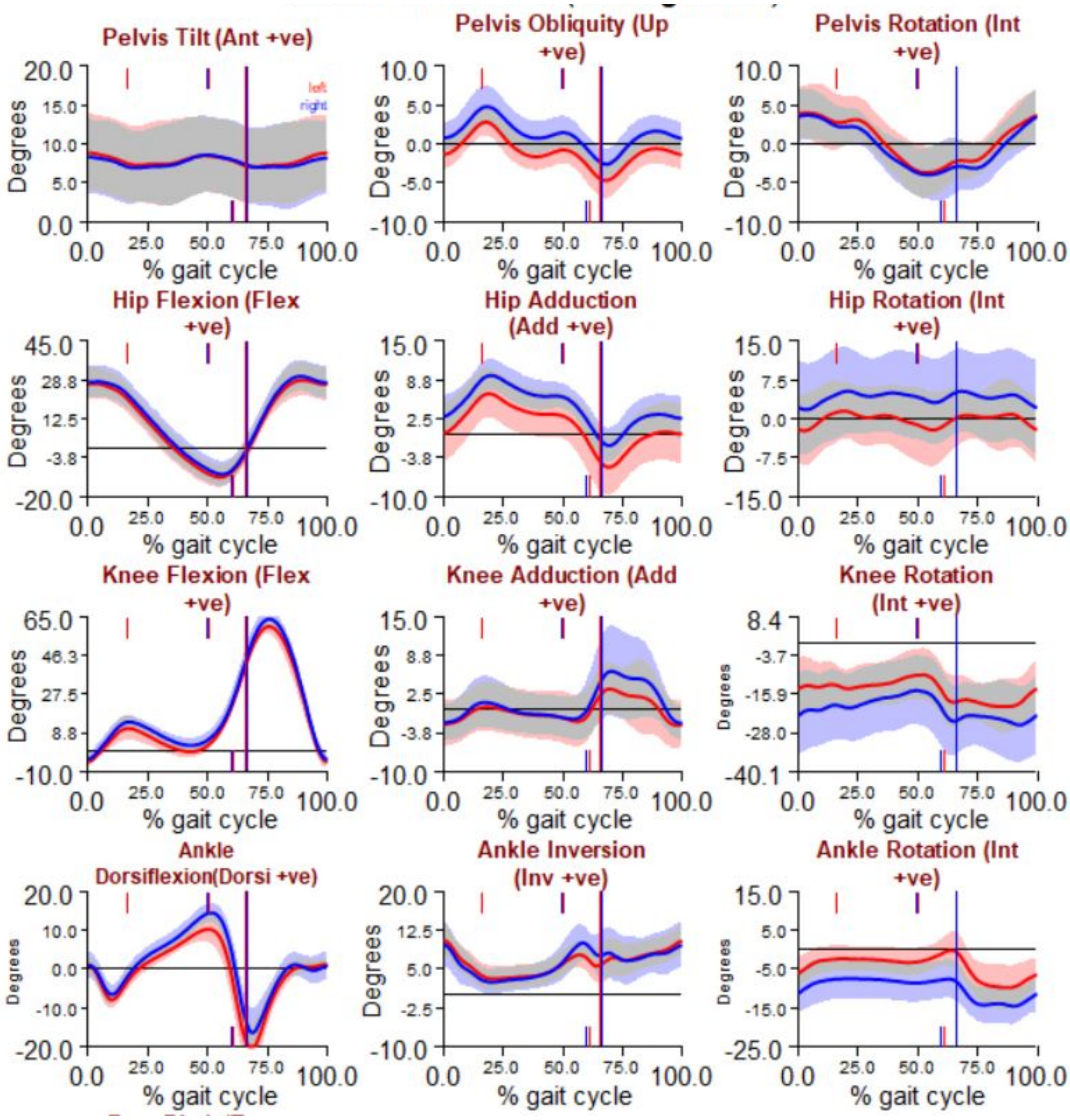

Figure 6S. Average normalized kinematic curves for group with leg length discrepancy >1cm without orthotic insoles during the gait cycle. The blue curves represent the data for the right leg (long leg) and the red curves the data for the left leg (short leg). Between-curve standard deviation is shaded in blue for the right leg and in red for the left leg. The stance phase and the swing phase are separated by the vertical line in blue.

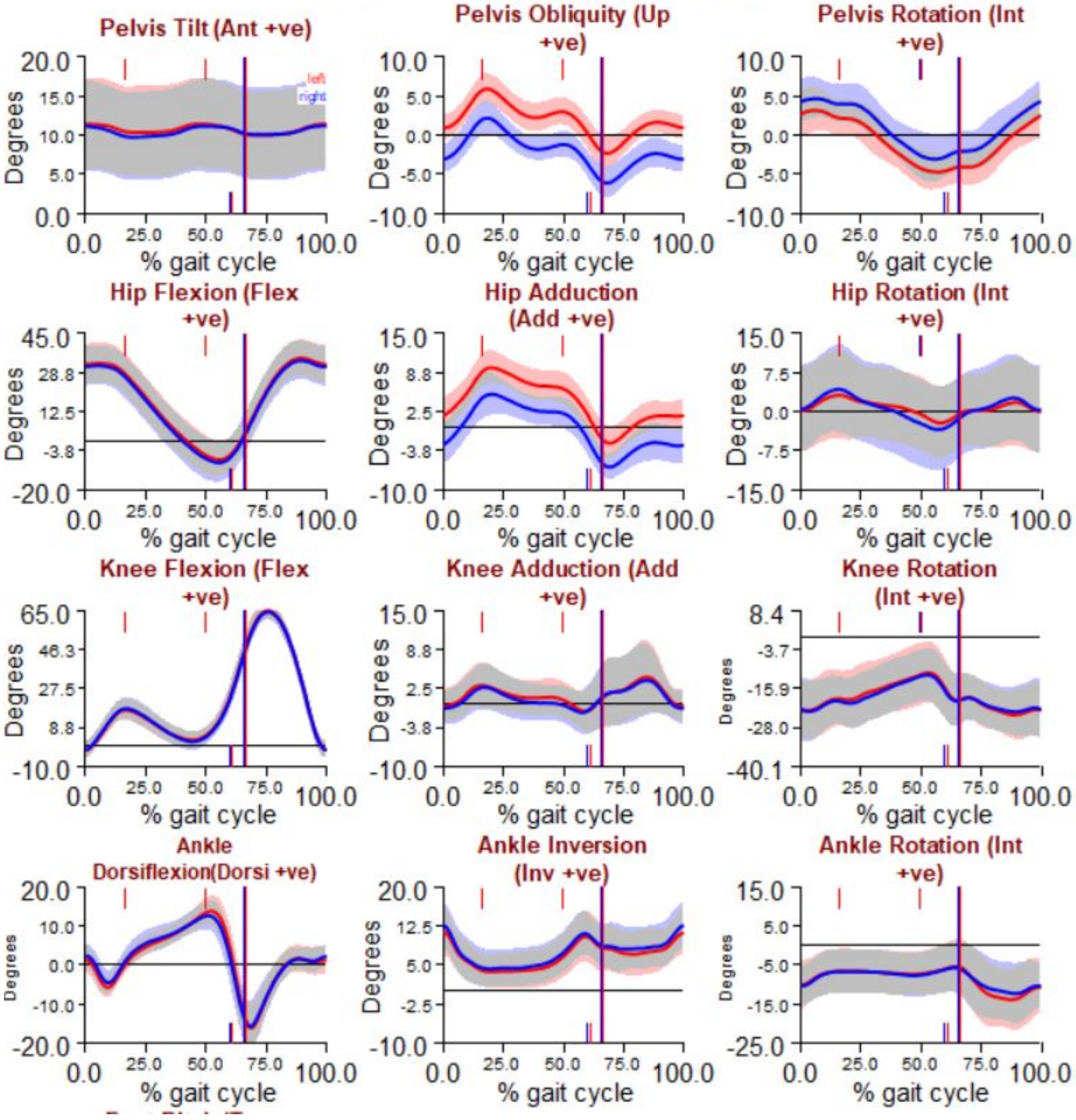

Figure 7S. Average normalized kinematic curves for group with leg length discrepancy >1cm with orthotic insoles during the gait cycle. The blue curves represent the data for the right leg (short leg) and the red curves the data for the left leg (long leg). Between-curve standard deviation is shaded in blue for the right leg and in red for the left leg. The stance phase and the swing phase are separated by the vertical line in blue.

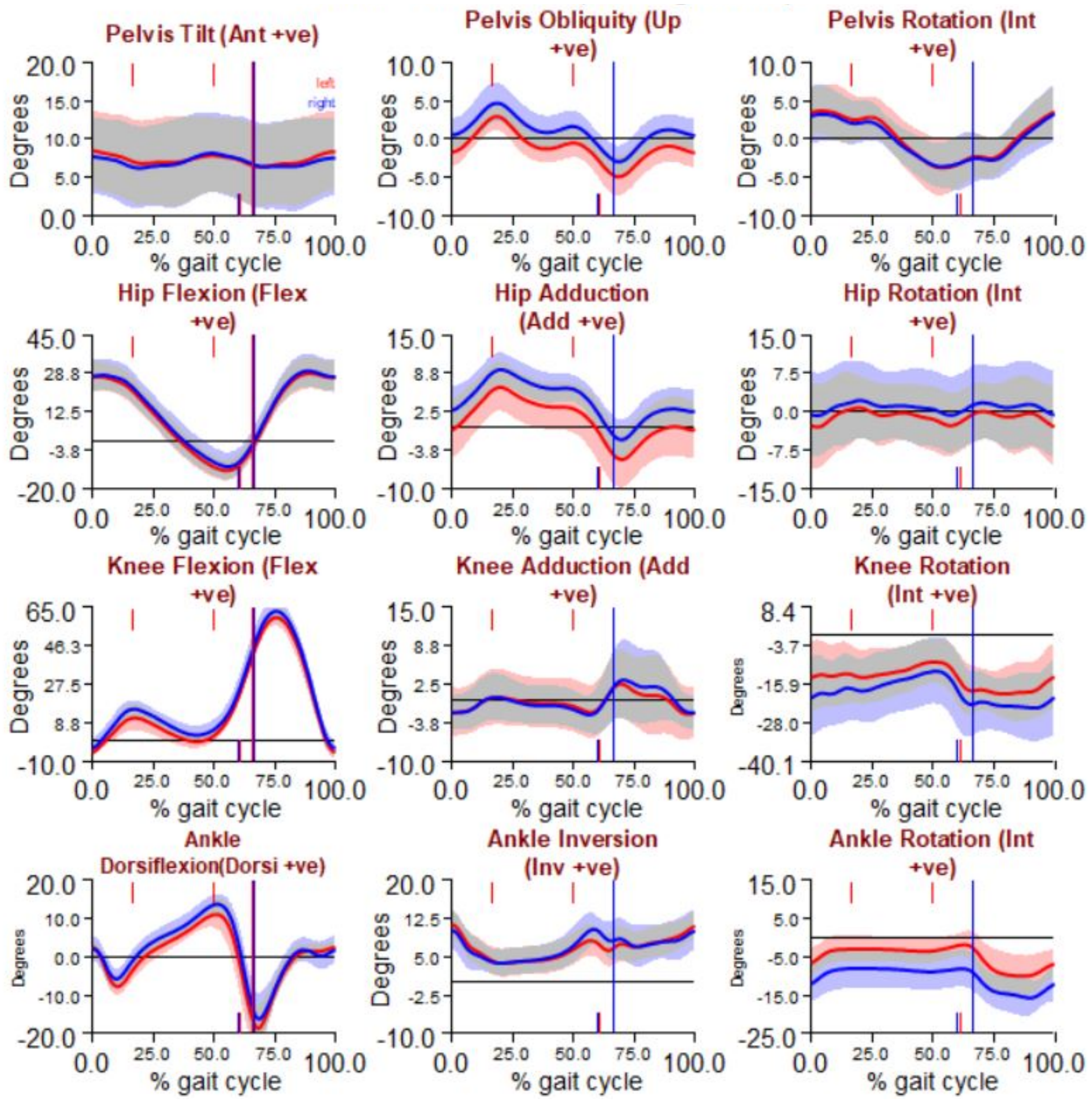

Figure 8S. Average normalized kinematic curves for group with leg length discrepancy >1cm with orthotic insoles during the gait cycle. The blue curves represent the data for the right leg (long leg) and the red curves the data for the left leg (short leg). Between-curve standard deviation is shaded in blue for the right leg and in red for the left leg. The stance phase and the swing phase are separated by the vertical line in blue.
